# Supplementary material for: A Novel Ultrasonographic Anthropometric-Independent Measurement of Median Nerve Swelling in Carpal Tunnel Syndrome: The “Nerve/Tendon Ratio” (NTR)
Source: Diagnostics (Basel). 2022 Oct 28;12(11):2621. doi: 10.3390/diagnostics12112621 (PMC9689936; doi:10.3390/diagnostics12112621)
Supplement: Supplementary file 1 [file diagnostics-12-02621-s001.zip › Figure S1-Correlation matrix (Spearman).pdf]

Correlation Matrix

Correlation Matrix

		MN-CSA	NTR	BMI	Height (cm)	Wrist circumf.	SCV sens.conduct.vel.	ML Motor Latency	Padua>3	Padua Scale
MN-CSA	Spearman's rho	—								
	p-value	—								
NTR	Spearman's rho	0.515 ***	—							
	p-value	< .001	—							
BMI	Spearman's rho	0.050	-0.161	—						
	p-value	0.674	0.170	—						
Height (cm)	Spearman's rho	0.120	-0.171	0.262 *	—					
	p-value	0.308	0.146	0.024	—					
Wrist circumf.	Spearman's rho	0.159	-0.127	0.463 ***	0.749 ***	—				
	p-value	0.177	0.282	< .001	< .001	—				
SCV sens.conduct.vel.	Spearman's rho	-0.550 ***	-0.611 ***	0.030	-0.032	-0.074	—			
	p-value	< .001	< .001	0.800	0.788	0.528	—			
ML Motor Latency	Spearman's rho	0.468 ***	0.558 ***	-0.010	0.189	0.278 *	-0.898 ***	—		
	p-value	< .001	< .001	0.934	0.120	0.021	< .001	—		
Padua>3	Spearman's rho	0.570 ***	0.543 ***	-0.026	0.012	0.088	-0.763 ***	0.653 ***	—	
	p-value	< .001	< .001	0.825	0.917	0.454	< .001	< .001	—	
Padua Scale	Spearman's rho	0.636 ***	0.650 ***	-0.043	0.083	0.086	-0.930 ***	0.862 ***	0.793 ***	—
	p-value	< .001	< .001	0.717	0.481	0.464	< .001	< .001	< .001	—

Note. * p < .05, ** p < .01, *** p < .001
