# Supplementary material for: A Novel Ultrasonographic Anthropometric-Independent Measurement of Median Nerve Swelling in Carpal Tunnel Syndrome: The “Nerve/Tendon Ratio” (NTR)
Source: Diagnostics (Basel). 2022 Oct 28;12(11):2621. doi: 10.3390/diagnostics12112621 (PMC9689936; doi:10.3390/diagnostics12112621)
Supplement: Supplementary file 1 [file diagnostics-12-02621-s001.zip › Figure S2-Partial correlation (Spearman).pdf]

Partial Correlation

Partial Correlation

		NTR	MN-CSA	SCV sens.conduct.vel.	ML Motor Latency	Padua Scale
NTR	Spearman's rho	—				
	p-value	—				
MN-CSA	Spearman's rho	0.564 ***	—			
	p-value	< .001	—			
SCV sens.conduct.vel.	Spearman's rho	-0.623 ***	-0.558 ***	—		
	p-value	< .001	< .001	—		
ML Motor Latency	Spearman's rho	0.606 ***	0.470 ***	-0.910 ***	—	
	p-value	< .001	< .001	< .001	—	
Padua Scale	Spearman's rho	0.674 ***	0.642 ***	-0.932 ***	0.874 ***	—
	p-value	< .001	< .001	< .001	< .001	—

Note. controlling for 'Height (cm)', 'Wrist circumf.', and 'Wheight (Kg)'

Note. * p < .05, ** p < .01, *** p < .001
