# Supplementary material for: A Novel Ultrasonographic Anthropometric-Independent Measurement of Median Nerve Swelling in Carpal Tunnel Syndrome: The “Nerve/Tendon Ratio” (NTR)
Source: Diagnostics (Basel). 2022 Oct 28;12(11):2621. doi: 10.3390/diagnostics12112621 (PMC9689936; doi:10.3390/diagnostics12112621)
Supplement: Supplementary file 1 [file diagnostics-12-02621-s001.zip › Figure S3-ANOVA (DSCF) MN-CSA and NTR in Padua stages.pdf]

One-Way ANOVA (Non-parametric)

Kruskal-Wallis

	χ^2	df	p
NTR	32.6	5	< .001
MN-CSA	32.8	5	< .001

Dwass-Steel-Critchlow-Fligner pairwise comparisons

Pairwise comparisons - NTR

		W	p
0	1	1.90	0.762
0	2	2.95	0.294
0	3	3.77	0.082
0	4	3.71	0.092
0	5	3.29	0.185
1	2	0.00	1.000
1	3	1.58	0.873
1	4	2.11	0.671
1	5	2.12	0.665
2	3	3.54	0.123
2	4	4.98	0.006
2	5	5.07	0.005
3	4	3.38	0.161
3	5	4.96	0.006
4	5	2.98	0.285

Pairwise comparisons - MN-CSA

		W	p
0	1	0.632	0.998
0	2	2.466	0.503
0	3	3.343	0.169
0	4	3.522	0.127
0	5	3.300	0.181
1	2	1.478	0.903
1	3	2.027	0.707
1	4	2.283	0.589
1	5	2.141	0.656
2	3	2.586	0.447
2	4	4.935	0.006
2	5	5.290	0.003
3	4	3.810	0.076
3	5	5.367	0.002
4	5	3.664	0.100
