# Supplementary material for: A Novel Ultrasonographic Anthropometric-Independent Measurement of Median Nerve Swelling in Carpal Tunnel Syndrome: The “Nerve/Tendon Ratio” (NTR)
Source: Diagnostics (Basel). 2022 Oct 28;12(11):2621. doi: 10.3390/diagnostics12112621 (PMC9689936; doi:10.3390/diagnostics12112621)
Supplement: Supplementary file 1 [file diagnostics-12-02621-s001.zip › Figure S4-Binomial regression analysis-ROC curves.pdf]

Binomial regression analysis between US parameters (both MN-CSA and NTR) vs diagnostic gold standard (both clinical CTS and EDS-defined severe CTS). The best cut-off (derived from Youden j-Statistic) is marked in **bold**, whereas the cut-off with the best positive predictive value is marker in ***bold italics***, all along the tables.

- MN-CSA vs CTS-clinical diagnosis (as gold standard)

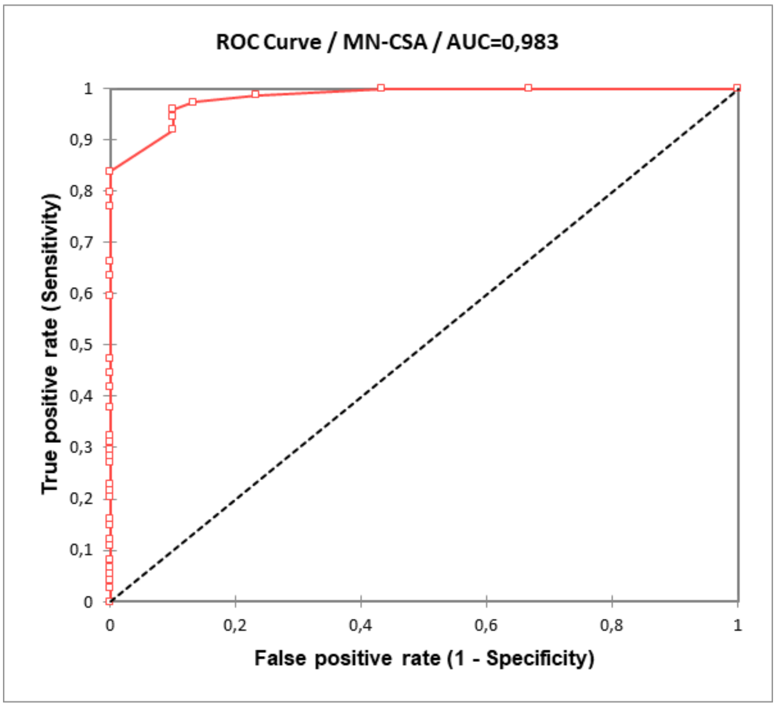

MN-CSA	Sensitivity	Lower bound (95%)	Upper bound (95%)	Specificity	Lower bound (95%)	Upper bound (95%)	PPV	NPV	LR+	LR-	Sensitivity+Specificity	Accuracy
4,000	1,000	0,939	1,000	0,333	0,192	0,514	0,787	1,000	1,500	0,000	1,333	0,808
5,000	1,000	0,939	1,000	0,567	0,392	0,726	0,851	1,000	2,308	0,000	1,567	0,875
6,000	0,986	0,919	1,000	0,767	0,587	0,884	0,913	0,958	4,228	0,018	1,753	0,923
7,000	0,973	0,900	0,998	0,867	0,695	0,952	0,947	0,929	7,297	0,031	1,840	0,942
**8,000**	**0,959**	**0,882**	**0,990**	**0,900**	**0,734**	**0,972**	**0,959**	**0,900**	**9,595**	**0,045**	**1,859**	**0,942**
8,300	0,946	0,864	0,982	0,900	0,734	0,972	0,959	0,871	9,459	0,060	1,846	0,933
8,600	0,919	0,830	0,965	0,900	0,734	0,972	0,958	0,818	9,189	0,090	1,819	0,913
9,000	***0,838***	***0,735***	***0,906***	***1,000***	***0,862***	***1,000***	***1,000***	***0,714***	***+Inf***	***0,162***	***1,838***	***0,885***
9,300	0,797	0,690	0,874	1,000	0,862	1,000	1,000	0,667	+Inf	0,203	1,797	0,856
9,600	0,770	0,661	0,852	1,000	0,862	1,000	1,000	0,638	+Inf	0,230	1,770	0,837
10,000	0,662	0,548	0,759	1,000	0,862	1,000	1,000	0,545	+Inf	0,338	1,662	0,760
10,300	0,635	0,521	0,735	1,000	0,862	1,000	1,000	0,526	+Inf	0,365	1,635	0,740
10,600	0,595	0,481	0,699	1,000	0,862	1,000	1,000	0,500	+Inf	0,405	1,595	0,712
11,000	0,473	0,364	0,585	1,000	0,862	1,000	1,000	0,435	+Inf	0,527	1,473	0,625
11,300	0,446	0,338	0,559	1,000	0,862	1,000	1,000	0,423	+Inf	0,554	1,446	0,606
11,500	0,419	0,313	0,533	1,000	0,862	1,000	1,000	0,411	+Inf	0,581	1,419	0,587
11,600	0,378	0,277	0,493	1,000	0,862	1,000	1,000	0,395	+Inf	0,622	1,378	0,558
12,000	0,324	0,229	0,438	1,000	0,862	1,000	1,000	0,375	+Inf	0,676	1,324	0,519

12,300	0,311	0,217	0,424	1,000	0,862	1,000	1,000	0,370	+Inf	0,689	1,311	0,510
12,600	0,297	0,205	0,410	1,000	0,862	1,000	1,000	0,366	+Inf	0,703	1,297	0,500
13,000	0,284	0,194	0,396	1,000	0,862	1,000	1,000	0,361	+Inf	0,716	1,284	0,490
13,300	0,270	0,182	0,382	1,000	0,862	1,000	1,000	0,357	+Inf	0,730	1,270	0,481
14,000	0,230	0,148	0,339	1,000	0,862	1,000	1,000	0,345	+Inf	0,770	1,230	0,452
14,300	0,216	0,137	0,324	1,000	0,862	1,000	1,000	0,341	+Inf	0,784	1,216	0,442
14,600	0,203	0,126	0,310	1,000	0,862	1,000	1,000	0,337	+Inf	0,797	1,203	0,433
15,000	0,162	0,094	0,265	1,000	0,862	1,000	1,000	0,326	+Inf	0,838	1,162	0,404
15,300	0,149	0,084	0,249	1,000	0,862	1,000	1,000	0,323	+Inf	0,851	1,149	0,394
16,000	0,122	0,064	0,218	1,000	0,862	1,000	1,000	0,316	+Inf	0,878	1,122	0,375
16,600	0,108	0,054	0,202	1,000	0,862	1,000	1,000	0,313	+Inf	0,892	1,108	0,365
17,000	0,081	0,035	0,170	1,000	0,862	1,000	1,000	0,306	+Inf	0,919	1,081	0,346
17,300	0,068	0,026	0,153	1,000	0,862	1,000	1,000	0,303	+Inf	0,932	1,068	0,337
19,000	0,054	0,018	0,136	1,000	0,862	1,000	1,000	0,300	+Inf	0,946	1,054	0,327
19,600	0,041	0,010	0,118	1,000	0,862	1,000	1,000	0,297	+Inf	0,959	1,041	0,317
21,000	0,027	0,002	0,100	1,000	0,862	1,000	1,000	0,294	+Inf	0,973	1,027	0,308
22,000	0,000	0,000	0,061	1,000	0,862	1,000		0,288		1,000	1,000	0,288

AUC	Standard error	Lower bound (95%)	Upper bound (95%)
0,983	0,009	0,966	1,000

Difference	0,483
z (Observed value)	56,251
z (Critical value)	1,960
p-value (Two-tailed)	< 0,0001
alpha	0,05

- NTR vs CTS-clinical diagnosis (as gold standard)

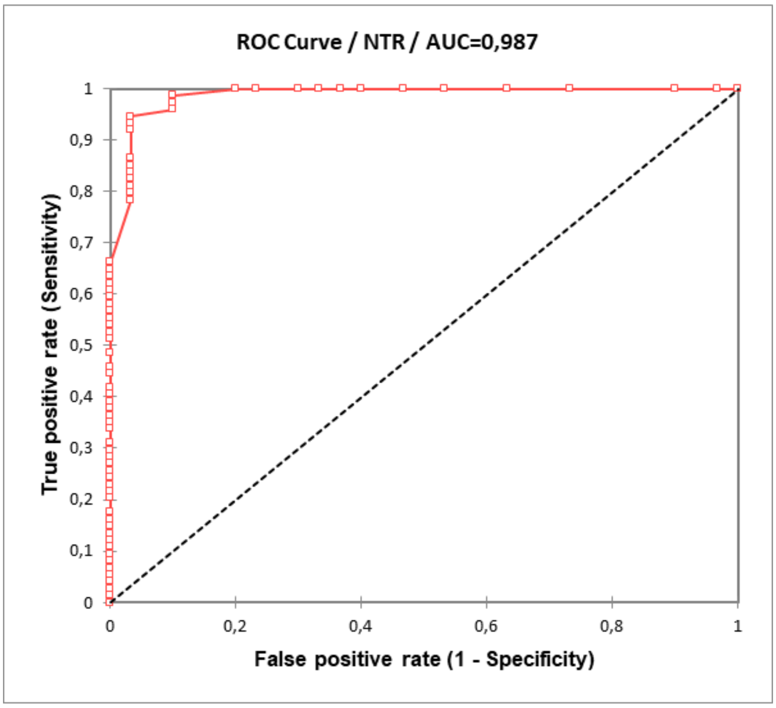

NTR	Sensitivity	Lower bound (95%)	Upper bound (95%)	Specificity	Lower bound (95%)	Upper bound (95%)	PPV	NPV	LR+	LR-	Sensitivity+Specificity	Accuracy
41,670	1,000	0,939	1,000	0,033	0,000	0,184	0,718	1,000	1,034	0,000	1,033	0,721
44,440	1,000	0,939	1,000	0,100	0,028	0,266	0,733	1,000	1,111	0,000	1,100	0,740
50,000	1,000	0,939	1,000	0,267	0,141	0,447	0,771	1,000	1,364	0,000	1,267	0,788
55,560	1,000	0,939	1,000	0,367	0,219	0,546	0,796	1,000	1,579	0,000	1,367	0,817
57,140	1,000	0,939	1,000	0,467	0,303	0,638	0,822	1,000	1,875	0,000	1,467	0,846
60,000	1,000	0,939	1,000	0,533	0,362	0,697	0,841	1,000	2,143	0,000	1,533	0,865
62,500	1,000	0,939	1,000	0,600	0,423	0,754	0,860	1,000	2,500	0,000	1,600	0,885
63,640	1,000	0,939	1,000	0,633	0,454	0,781	0,871	1,000	2,727	0,000	1,633	0,894
66,670	1,000	0,939	1,000	0,667	0,486	0,808	0,881	1,000	3,000	0,000	1,667	0,904
71,430	1,000	0,939	1,000	0,700	0,519	0,834	0,892	1,000	3,333	0,000	1,700	0,913
72,000	1,000	0,939	1,000	0,767	0,587	0,884	0,914	1,000	4,286	0,000	1,767	0,933
72,730	1,000	0,939	1,000	0,800	0,622	0,907	0,925	1,000	5,000	0,000	1,800	0,942
75,000	0,986	0,919	1,000	0,900	0,734	0,972	0,961	0,964	9,865	0,015	1,886	0,962
76,900	0,973	0,900	0,998	0,900	0,734	0,972	0,960	0,931	9,730	0,030	1,873	0,952
82,800	0,959	0,882	0,990	0,900	0,734	0,972	0,959	0,900	9,595	0,045	1,859	0,942
**83,000**	**0,946**	**0,864**	**0,982**	**0,967**	**0,816**	**1,000**	**0,986**	**0,879**	**28,378**	**0,056**	**1,913**	**0,952**
85,700	0,932	0,847	0,974	0,967	0,816	1,000	0,986	0,853	27,973	0,070	1,899	0,942
89,200	0,919	0,830	0,965	0,967	0,816	1,000	0,986	0,829	27,568	0,084	1,886	0,933
90,000	0,865	0,766	0,926	0,967	0,816	1,000	0,985	0,744	25,946	0,140	1,832	0,894
92,200	0,851	0,751	0,916	0,967	0,816	1,000	0,984	0,725	25,541	0,154	1,818	0,885
92,300	0,838	0,735	0,906	0,967	0,816	1,000	0,984	0,707	25,135	0,168	1,805	0,875
93,000	0,824	0,720	0,895	0,967	0,816	1,000	0,984	0,690	24,730	0,182	1,791	0,865
95,000	0,811	0,705	0,884	0,967	0,816	1,000	0,984	0,674	24,324	0,196	1,777	0,856

96,000	0,797	0,690	0,874	0,967	0,816	1,000	0,983	0,659	23,919	0,210	1,764	0,846
96,360	0,784	0,676	0,863	0,967	0,816	1,000	0,983	0,644	23,514	0,224	1,750	0,837
100,000	***0,662***	***0,548***	***0,759***	***1,000***	***0,862***	***1,000***	***1,000***	***0,545***	***+Inf***	***0,338***	***1,662***	***0,760***
102,100	0,649	0,535	0,747	1,000	0,862	1,000	1,000	0,536	+Inf	0,351	1,649	0,750
102,720	0,635	0,521	0,735	1,000	0,862	1,000	1,000	0,526	+Inf	0,365	1,635	0,740
103,000	0,622	0,507	0,723	1,000	0,862	1,000	1,000	0,517	+Inf	0,378	1,622	0,731
103,300	0,608	0,494	0,711	1,000	0,862	1,000	1,000	0,508	+Inf	0,392	1,608	0,721
104,000	0,595	0,481	0,699	1,000	0,862	1,000	1,000	0,500	+Inf	0,405	1,595	0,712
105,400	0,581	0,467	0,687	1,000	0,862	1,000	1,000	0,492	+Inf	0,419	1,581	0,702
106,000	0,568	0,454	0,674	1,000	0,862	1,000	1,000	0,484	+Inf	0,432	1,568	0,692
106,600	0,554	0,441	0,662	1,000	0,862	1,000	1,000	0,476	+Inf	0,446	1,554	0,683
107,600	0,541	0,428	0,649	1,000	0,862	1,000	1,000	0,469	+Inf	0,459	1,541	0,673
107,690	0,527	0,415	0,636	1,000	0,862	1,000	1,000	0,462	+Inf	0,473	1,527	0,663
109,100	0,514	0,402	0,624	1,000	0,862	1,000	1,000	0,455	+Inf	0,486	1,514	0,654
110,000	0,486	0,376	0,598	1,000	0,862	1,000	1,000	0,441	+Inf	0,514	1,486	0,635
111,000	0,459	0,351	0,572	1,000	0,862	1,000	1,000	0,429	+Inf	0,541	1,459	0,615
111,800	0,446	0,338	0,559	1,000	0,862	1,000	1,000	0,423	+Inf	0,554	1,446	0,606
112,500	0,419	0,313	0,533	1,000	0,862	1,000	1,000	0,411	+Inf	0,581	1,419	0,587
113,000	0,405	0,301	0,519	1,000	0,862	1,000	1,000	0,405	+Inf	0,595	1,405	0,577
114,400	0,392	0,289	0,506	1,000	0,862	1,000	1,000	0,400	+Inf	0,608	1,392	0,567
115,000	0,378	0,277	0,493	1,000	0,862	1,000	1,000	0,395	+Inf	0,622	1,378	0,558
116,000	0,365	0,265	0,479	1,000	0,862	1,000	1,000	0,390	+Inf	0,635	1,365	0,548
116,200	0,351	0,253	0,465	1,000	0,862	1,000	1,000	0,385	+Inf	0,649	1,351	0,538
116,250	0,338	0,241	0,452	1,000	0,862	1,000	1,000	0,380	+Inf	0,662	1,338	0,529
120,000	0,311	0,217	0,424	1,000	0,862	1,000	1,000	0,370	+Inf	0,689	1,311	0,510
120,900	0,297	0,205	0,410	1,000	0,862	1,000	1,000	0,366	+Inf	0,703	1,297	0,500
122,000	0,284	0,194	0,396	1,000	0,862	1,000	1,000	0,361	+Inf	0,716	1,284	0,490
122,200	0,270	0,182	0,382	1,000	0,862	1,000	1,000	0,357	+Inf	0,730	1,270	0,481
122,800	0,257	0,171	0,368	1,000	0,862	1,000	1,000	0,353	+Inf	0,743	1,257	0,471
126,000	0,243	0,160	0,353	1,000	0,862	1,000	1,000	0,349	+Inf	0,757	1,243	0,462
128,500	0,230	0,148	0,339	1,000	0,862	1,000	1,000	0,345	+Inf	0,770	1,230	0,452
133,000	0,216	0,137	0,324	1,000	0,862	1,000	1,000	0,341	+Inf	0,784	1,216	0,442
133,300	0,203	0,126	0,310	1,000	0,862	1,000	1,000	0,337	+Inf	0,797	1,203	0,433
136,300	0,176	0,105	0,280	1,000	0,862	1,000	1,000	0,330	+Inf	0,824	1,176	0,413
137,500	0,162	0,094	0,265	1,000	0,862	1,000	1,000	0,326	+Inf	0,838	1,162	0,404
139,000	0,149	0,084	0,249	1,000	0,862	1,000	1,000	0,323	+Inf	0,851	1,149	0,394

142,000	0,135	0,074	0,234	1,000	0,862	1,000	1,000	0,319	+Inf	0,865	1,135	0,385
146,000	0,122	0,064	0,218	1,000	0,862	1,000	1,000	0,316	+Inf	0,878	1,122	0,375
154,000	0,108	0,054	0,202	1,000	0,862	1,000	1,000	0,313	+Inf	0,892	1,108	0,365
160,000	0,095	0,044	0,186	1,000	0,862	1,000	1,000	0,309	+Inf	0,905	1,095	0,356
166,000	0,081	0,035	0,170	1,000	0,862	1,000	1,000	0,306	+Inf	0,919	1,081	0,346
170,000	0,068	0,026	0,153	1,000	0,862	1,000	1,000	0,303	+Inf	0,932	1,068	0,337
172,000	0,054	0,018	0,136	1,000	0,862	1,000	1,000	0,300	+Inf	0,946	1,054	0,327
173,000	0,041	0,010	0,118	1,000	0,862	1,000	1,000	0,297	+Inf	0,959	1,041	0,317
196,000	0,027	0,002	0,100	1,000	0,862	1,000	1,000	0,294	+Inf	0,973	1,027	0,308
210,000	0,014	0,000	0,081	1,000	0,862	1,000	1,000	0,291	+Inf	0,986	1,014	0,298
220,000	0,000	0,000	0,061	1,000	0,862	1,000		0,288		1,000	1,000	0,288

AUC	Standard error	Lower bound (95%)	Upper bound (95%)
0,987	0,010	0,968	1,000

Difference	0,487
z (Observed value)	50,470
z (Critical value)	1,960
p-value (Two-tailed)	< 0,0001
alpha	0,05

- MN-CSA vs severe CTS (EDS-defined,Padua Scale>3) (as gold standard)

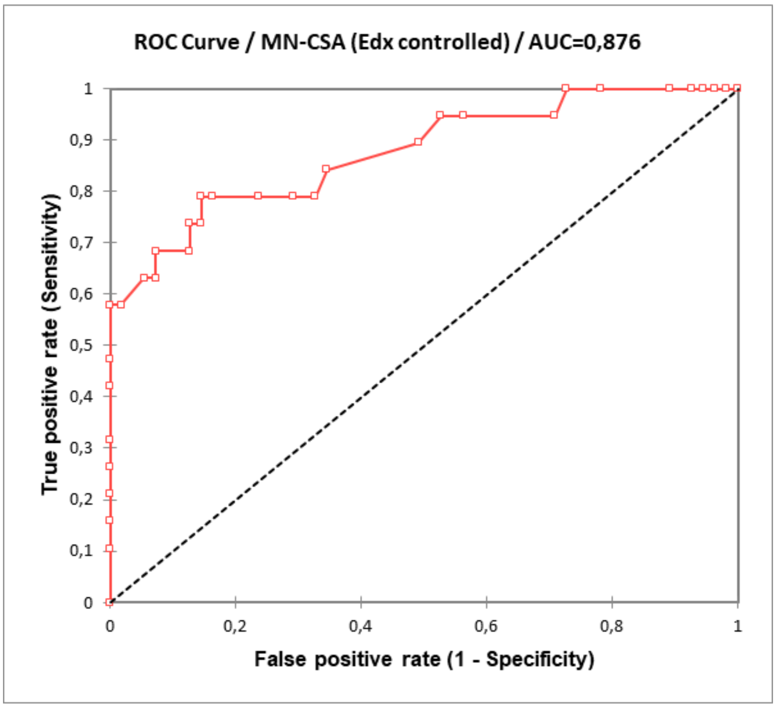

MN-CSA (EDS controlled)	Sensitivity	Lower bound (95%)	Upper bound (95%)	Specificity	Lower bound (95%)	Upper bound (95%)	PPV	NPV	LR+	LR-	Sensitivity+Specificity	Accuracy
6,000	1,000	0,798	1,000	0,018	0,000	0,107	0,260	1,000	1,019	0,000	1,018	0,270
7,000	1,000	0,798	1,000	0,036	0,004	0,132	0,264	1,000	1,038	0,000	1,036	0,284
8,000	1,000	0,798	1,000	0,055	0,014	0,156	0,268	1,000	1,058	0,000	1,055	0,297
8,300	1,000	0,798	1,000	0,073	0,025	0,179	0,271	1,000	1,078	0,000	1,073	0,311
8,600	1,000	0,798	1,000	0,109	0,048	0,223	0,279	1,000	1,122	0,000	1,109	0,338
9,000	1,000	0,798	1,000	0,218	0,129	0,346	0,306	1,000	1,279	0,000	1,218	0,419
9,300	1,000	0,798	1,000	0,273	0,173	0,404	0,322	1,000	1,375	0,000	1,273	0,459
9,600	0,947	0,732	1,000	0,291	0,188	0,423	0,316	0,941	1,336	0,181	1,238	0,459
10,000	0,947	0,732	1,000	0,436	0,314	0,567	0,367	0,960	1,681	0,121	1,384	0,568
10,300	0,947	0,732	1,000	0,473	0,347	0,602	0,383	0,963	1,797	0,111	1,420	0,595
10,600	0,895	0,671	0,981	0,509	0,381	0,636	0,386	0,933	1,823	0,207	1,404	0,608
11,000	0,842	0,614	0,951	0,655	0,522	0,766	0,457	0,923	2,438	0,241	1,497	0,703
11,300	0,789	0,560	0,919	0,673	0,540	0,782	0,455	0,902	2,412	0,313	1,462	0,703
11,500	0,789	0,560	0,919	0,709	0,577	0,812	0,484	0,907	2,714	0,297	1,499	0,730
11,600	0,789	0,560	0,919	0,764	0,635	0,857	0,536	0,913	3,340	0,276	1,553	0,770
12,000	0,789	0,560	0,919	0,836	0,714	0,913	0,625	0,920	4,825	0,252	1,626	0,824
**12,300**	**0,789**	**0,560**	**0,919**	**0,855**	**0,735**	**0,926**	**0,652**	**0,922**	**5,428**	**0,246**	**1,644**	**0,838**
12,600	0,737	0,508	0,884	0,855	0,735	0,926	0,636	0,904	5,066	0,308	1,591	0,824
13,000	0,737	0,508	0,884	0,873	0,756	0,939	0,667	0,906	5,789	0,302	1,610	0,838
13,300	0,684	0,458	0,847	0,873	0,756	0,939	0,650	0,889	5,376	0,362	1,557	0,824
14,000	0,684	0,458	0,847	0,927	0,821	0,975	0,765	0,895	9,408	0,341	1,611	0,865
14,300	0,632	0,409	0,808	0,927	0,821	0,975	0,750	0,879	8,684	0,397	1,559	0,851
14,600	0,632	0,409	0,808	0,945	0,844	0,986	0,800	0,881	11,579	0,390	1,577	0,865

15,000	0,579	0,363	0,768	0,982	0,893	1,000	0,917	0,871	31,842	0,429	1,561	0,878
15,300	***0,579***	***0,363***	***0,768***	***1,000***	***0,920***	***1,000***	***1,000***	***0,873***	***+Inf***	***0,421***	***1,579***	***0,892***
16,000	0,474	0,274	0,682	1,000	0,920	1,000	1,000	0,846	+Inf	0,526	1,474	0,865
16,600	0,421	0,232	0,637	1,000	0,920	1,000	1,000	0,833	+Inf	0,579	1,421	0,851
17,000	0,316	0,153	0,542	1,000	0,920	1,000	1,000	0,809	+Inf	0,684	1,316	0,824
17,300	0,263	0,116	0,492	1,000	0,920	1,000	1,000	0,797	+Inf	0,737	1,263	0,811
19,000	0,211	0,081	0,440	1,000	0,920	1,000	1,000	0,786	+Inf	0,789	1,211	0,797
19,600	0,158	0,049	0,386	1,000	0,920	1,000	1,000	0,775	+Inf	0,842	1,158	0,784
21,000	0,105	0,019	0,329	1,000	0,920	1,000	1,000	0,764	+Inf	0,895	1,105	0,770
22,000	0,000	0,000	0,202	1,000	0,920	1,000		0,743		1,000	1,000	0,743

AUC	Standard error	Lower bound (95%)	Upper bound (95%)
0,876	0,051	0,776	0,975

Difference	0,376
z (Observed value)	7,372
z (Critical value)	1,960
p-value (Two-tailed)	< 0,0001
alpha	0,05

- NTR vs severe CTS (EDS-defined,Padua Scale>3) (as gold standard)

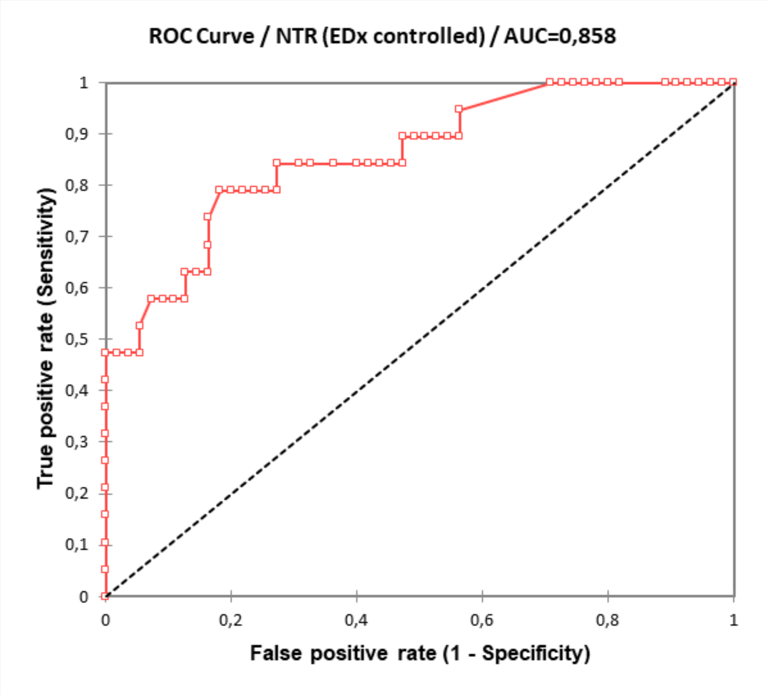

NTR (EDx controlled)	Sensitivity	Lower bound (95%)	Upper bound (95%)	Specificity	Lower bound (95%)	Upper bound (95%)	PPV	NPV	LR+	LR-	Sensitivity+Specificity	Accuracy
75,000	1,000	0,798	1,000	0,018	0,000	0,107	0,260	1,000	1,019	0,000	1,018	0,270
76,900	1,000	0,798	1,000	0,036	0,004	0,132	0,264	1,000	1,038	0,000	1,036	0,284
82,800	1,000	0,798	1,000	0,055	0,014	0,156	0,268	1,000	1,058	0,000	1,055	0,297
83,000	1,000	0,798	1,000	0,073	0,025	0,179	0,271	1,000	1,078	0,000	1,073	0,311
85,700	1,000	0,798	1,000	0,091	0,036	0,201	0,275	1,000	1,100	0,000	1,091	0,324
89,200	1,000	0,798	1,000	0,109	0,048	0,223	0,279	1,000	1,122	0,000	1,109	0,338
90,000	1,000	0,798	1,000	0,182	0,101	0,306	0,297	1,000	1,222	0,000	1,182	0,392
92,200	1,000	0,798	1,000	0,200	0,115	0,326	0,302	1,000	1,250	0,000	1,200	0,405
92,300	1,000	0,798	1,000	0,218	0,129	0,346	0,306	1,000	1,279	0,000	1,218	0,419
93,000	1,000	0,798	1,000	0,236	0,143	0,365	0,311	1,000	1,310	0,000	1,236	0,432
95,000	1,000	0,798	1,000	0,255	0,158	0,385	0,317	1,000	1,341	0,000	1,255	0,446
96,000	1,000	0,798	1,000	0,273	0,173	0,404	0,322	1,000	1,375	0,000	1,273	0,459
96,360	1,000	0,798	1,000	0,291	0,188	0,423	0,328	1,000	1,410	0,000	1,291	0,473
100,000	0,947	0,732	1,000	0,436	0,314	0,567	0,367	0,960	1,681	0,121	1,384	0,568
102,100	0,895	0,671	0,981	0,436	0,314	0,567	0,354	0,923	1,587	0,241	1,331	0,554
102,720	0,895	0,671	0,981	0,455	0,331	0,585	0,362	0,926	1,640	0,232	1,349	0,568
103,000	0,895	0,671	0,981	0,473	0,347	0,602	0,370	0,929	1,697	0,223	1,367	0,581
103,300	0,895	0,671	0,981	0,491	0,364	0,619	0,378	0,931	1,758	0,214	1,386	0,595
104,000	0,895	0,671	0,981	0,509	0,381	0,636	0,386	0,933	1,823	0,207	1,404	0,608
105,400	0,895	0,671	0,981	0,527	0,398	0,653	0,395	0,935	1,893	0,200	1,422	0,622
106,000	0,842	0,614	0,951	0,527	0,398	0,653	0,381	0,906	1,781	0,299	1,369	0,608

106,600	0,842	0,614	0,951	0,545	0,415	0,669	0,390	0,909	1,853	0,289	1,388	0,622
107,600	0,842	0,614	0,951	0,564	0,433	0,686	0,400	0,912	1,930	0,280	1,406	0,635
107,690	0,842	0,614	0,951	0,582	0,450	0,702	0,410	0,914	2,014	0,271	1,424	0,649
109,100	0,842	0,614	0,951	0,600	0,468	0,719	0,421	0,917	2,105	0,263	1,442	0,662
110,000	0,842	0,614	0,951	0,636	0,504	0,751	0,444	0,921	2,316	0,248	1,478	0,689
111,000	0,842	0,614	0,951	0,673	0,540	0,782	0,471	0,925	2,573	0,235	1,515	0,716
111,800	0,842	0,614	0,951	0,691	0,559	0,797	0,485	0,927	2,724	0,229	1,533	0,730
112,500	0,842	0,614	0,951	0,727	0,596	0,827	0,516	0,930	3,088	0,217	1,569	0,757
113,000	0,789	0,560	0,919	0,727	0,596	0,827	0,500	0,909	2,895	0,289	1,517	0,743
114,400	0,789	0,560	0,919	0,745	0,615	0,842	0,517	0,911	3,102	0,282	1,535	0,757
115,000	0,789	0,560	0,919	0,764	0,635	0,857	0,536	0,913	3,340	0,276	1,553	0,770
116,000	0,789	0,560	0,919	0,782	0,654	0,871	0,556	0,915	3,618	0,269	1,571	0,784
116,200	0,789	0,560	0,919	0,800	0,674	0,885	0,577	0,917	3,947	0,263	1,589	0,797
**116,250**	**0,789**	**0,560**	**0,919**	**0,818**	**0,694**	**0,899**	**0,600**	**0,918**	**4,342**	**0,257**	**1,608**	**0,811**
120,000	0,737	0,508	0,884	0,836	0,714	0,913	0,609	0,902	4,503	0,315	1,573	0,811
120,900	0,684	0,458	0,847	0,836	0,714	0,913	0,591	0,885	4,181	0,378	1,521	0,797
122,000	0,632	0,409	0,808	0,836	0,714	0,913	0,571	0,868	3,860	0,441	1,468	0,784
122,200	0,632	0,409	0,808	0,855	0,735	0,926	0,600	0,870	4,342	0,431	1,486	0,797
122,800	0,632	0,409	0,808	0,873	0,756	0,939	0,632	0,873	4,962	0,422	1,504	0,811
126,000	0,579	0,363	0,768	0,873	0,756	0,939	0,611	0,857	4,549	0,482	1,452	0,797
128,500	0,579	0,363	0,768	0,891	0,777	0,952	0,647	0,860	5,307	0,473	1,470	0,811
133,000	0,579	0,363	0,768	0,909	0,799	0,964	0,688	0,862	6,368	0,463	1,488	0,824
133,300	0,579	0,363	0,768	0,927	0,821	0,975	0,733	0,864	7,961	0,454	1,506	0,838
136,300	0,526	0,318	0,726	0,945	0,844	0,986	0,769	0,852	9,649	0,501	1,472	0,838
137,500	0,474	0,274	0,682	0,945	0,844	0,986	0,750	0,839	8,684	0,557	1,419	0,824
139,000	0,474	0,274	0,682	0,964	0,868	0,996	0,818	0,841	13,026	0,546	1,437	0,838
142,000	0,474	0,274	0,682	0,982	0,893	1,000	0,900	0,844	26,053	0,536	1,456	0,851
146,000	***0,474***	***0,274***	***0,682***	***1,000***	***0,920***	***1,000***	***1,000***	***0,846***	***+Inf***	***0,526***	***1,474***	***0,865***
154,000	0,421	0,232	0,637	1,000	0,920	1,000	1,000	0,833	+Inf	0,579	1,421	0,851
160,000	0,368	0,192	0,591	1,000	0,920	1,000	1,000	0,821	+Inf	0,632	1,368	0,838
166,000	0,316	0,153	0,542	1,000	0,920	1,000	1,000	0,809	+Inf	0,684	1,316	0,824
170,000	0,263	0,116	0,492	1,000	0,920	1,000	1,000	0,797	+Inf	0,737	1,263	0,811
172,000	0,211	0,081	0,440	1,000	0,920	1,000	1,000	0,786	+Inf	0,789	1,211	0,797
173,000	0,158	0,049	0,386	1,000	0,920	1,000	1,000	0,775	+Inf	0,842	1,158	0,784
196,000	0,105	0,019	0,329	1,000	0,920	1,000	1,000	0,764	+Inf	0,895	1,105	0,770

210,000	0,053	0,000	0,268	1,000	0,920	1,000	1,000	0,753	+Inf	0,947	1,053	0,757
220,000	0,000	0,000	0,202	1,000	0,920	1,000		0,743		1,000	1,000	0,743

AUC	Standard error	Lower bound (95%)	Upper bound (95%)
0,858	0,051	0,759	0,958

Difference	0,358
z (Observed value)	7,060
z (Critical value)	1,960
p-value (Two-tailed)	< 0,0001
alpha	0,05
